# Learning from the past to design better trials in second-line treatment for mesothelioma patients

**DOI:** 10.3332/ecancer.2018.881

**Published:** 2018-11-12

**Authors:** Alfredo Addeo, Giuseppe Luigi Banna, Pierre-Yves Dietrich

**Affiliations:** 1 Oncology Department, University Hospital Geneva, 1205 Geneva, Switzerland; 2 Division of Medical Oncology, Cannizzaro Hospital, Via Messina 829, 95126, Catania, Italy

**Keywords:** mesothelioma, trial design, angiogenesis

## Abstract

Malignant pleural mesothelioma (MPM) is an aggressive cancer characterised by modest sensitivity to systemic chemotherapy. The standard treatment remains platinum-based chemotherapy with pemetrexed. Recently, the addition of an antiangiogenic drug, bevacizumab, to first-line chemotherapy has been shown to improve overall survival. All the patients, unfortunately, will progress, and currently, there is no standard treatment approved in second-line. Recently, the results of the NGR015 phase III randomised with NGR-hTNF plus chemotherapy versus placebo in addition to physician’s choice second-line chemotherapy for MPM have been published. Despite encouraging data achieved in previous phase I and phase II studies, the NGR-hTNF drug failed to meet the primary endpoint of the study, although a signal of activity was observed in the group of patients who had a shorter treatment failure interval from the first-line treatment.

Hereby, we start from this recent trial to highlight once more the importance of thoroughly investigating possible predictive factors during the early drug development phase to allow more efficient phase III trial design, thus avoiding the possibility of potentially effective molecules like NGR-hTNF, continuing to be wasted.

Malignant pleural mesothelioma (MPM) is an aggressive cancer characterised by modest sensitivity to systemic chemotherapy [1]. The addition of an antiangiogenic drug, bevacizumab, to first-line chemotherapy has been shown to improve overall survival (OS) in a large phase III trial from 16.1 months up to 18.8 months. Unfortunately, there is no standard second line yet approved for such patients. Therefore, well-designed clinical trials in the second-line setting are essential to meet this need.

Recently, the results of the NGR015 [2] phase III randomised with NGR-hTNF, a new drug which increases the intratumoural chemotherapy penetration and promotes the T cell infiltration by modifying the tumour microenvironment, versus placebo in addition to physician’s choice second-line chemotherapy for MPM have been reported. The study didn’t meet its primary endpoint OS in the intention to treat the population, but it suggested a possible clinically meaningful interaction in the subgroup of patients with a short treatment-free interval (TFI) from the first-line therapy, considering as its cutoff the median time observed in the *post-hoc* exploratory sensitivity analysis. It is not the first time that TFI has been shown to be predictive of better outcome in patients treated with antiangiogenic drugs in the second-line setting, as for example in non-small cell lung cancer (NSCLC) within the LUME-Lung trial by the addition of nintedanib to docetaxel [3].

Patients enrolled in clinical trials [4] should be balanced according to known potentially predictive factors by stratification. Even more, within the NGR015 trial, a proper stratification including, besides performance status (PS) and chemotherapy, the histology, BAP1 mutation and TFI might have shown different results. In fact, the histology was not used as a stratification factor despite being an independent prognostic factor along with PS in the previous phase II study [5]. TFI was investigated in the previous phase II trial [5], but as a continuous variable not as a dichotomous variable in order to identify a possible cutoff. This would have led to the adoption of it as a further stratification factor. As a consequence, a pre-specified analysis of OS could have produced more reliable and useful evidence. A good example where a properly planned stratification and subgroup analysis changed clinical practice has been the trial of Scagliotti *et al* [6] in NSCLC where histology was included among others stratification factors and a pre-specified analysis allowed the authors to show a clinically meaningful benefit of pemetrexed in adenocarcinoma patients.

As far as we know, all the ongoing trials in second-line MPM have not been properly addressing TFI in their trial design, raising the possibility of a negative impact on the final result.

The NGR015 trial showed once more that possible predictive factor should be properly explored and identified during the early drug development phase to allow more efficient phase III trial design.

## Conclusion

We have pointed out the importance of predictive factors and a proper stratification as the way to design successful trials. It is certainly concerning that, at the moment, no trials in the second-line treatment of MPM have been designed taking into account such factors and in particular the TFI.

This might, therefore, lead to further failures despite the possible efficacy and activity of the molecule under investigation.

## Conflicts of interest

Alfredo Addeo has received research funding from Boehringer Ingelheim. He has played a consulting or advisory role in relationship with SD Oncology, Bristol-Myers Squibb, Roche, AstraZeneca and Boehringer Ingelheim. Giuseppe L Banna has played a consulting or advisory role in relationship with Janssen-Cilag and Boehringer Ingelheim.

## Funding

This paper received no funding.
